# NCI State of the Workforce - Aging and Disabilities: Results and Implications for Policy & Practice

**DOI:** 10.1093/geroni/igaf122.1312

**Published:** 2025-12-31

**Authors:** Dorothy Hiersteiner, Lindsay Dubois, Rosa Plasencia

**Affiliations:** Human Services Research Institute (HSRI), Cambridge, Massachusetts, United States; Human Services Research Institute, Cambridge, Massachusetts, United States; ADvancing States, Arlington, Virginia, United States

## Abstract

There are approximately 5 million direct service workers (DSWs) providing supports to older adults and people with disabilities in the US. While this number seems high, the demand for DSWs often outstrips the supply; combined with high levels of turnover at direct care provider agencies, the workforce crisis is intensifying and has a substantial impact on the quality of supports. This presentation will showcase key findings from the National Core Indicators (NCI) State of the Workforce Aging and Disabilities 2023 survey of more than 1,200 provider agencies in 6 states. Altogether, these provider agencies employ more than 1.4 million DSWs. Among the states that participated, the average turnover ratio was 48%, with 1 out of ever 3 agencies reporting that they turned away or stopped accepting referrals due to staffing issues. The median hourly wage across states was $16.00 per hour, and 99% of agencies pay a median hourly wage that is more than $0.50 below living wage in their state. Finally, just 55% of agencies offered paid time off to DSWs, 41% offered health insurance, and 38% offered an employer-sponsored retirement plan. The low wages and limited benefits offered to DSWs are likely contributors to the high rates of turnover. These data highlight the unsustainable working conditions for many DSWs, and point to tangible quality improvement opportunities for state and service providers.

